# Arterial stiffness in adults with Long COVID in sub‐Saharan Africa

**DOI:** 10.14814/phy2.70029

**Published:** 2024-09-09

**Authors:** Chikopela Theresa, Bwalya Katebe, Cyndya A. Shibao, Annet Kirabo

**Affiliations:** ^1^ Department of Human Physiology, Faculty of Medicine Lusaka Apex Medical University Lusaka Zambia; ^2^ Department of Medicine Vanderbilt University Medical Center Nashville Tennessee USA

**Keywords:** arterial stiffness, long COVID, pulse wave velocity

## Abstract

Severe acute coronavirus‐2 (SARS‐CoV‐2) infection has been associated with endothelial damage, and impaired nitric oxide production, which results in arterial stiffness and increased risk of cardiovascular disease. Long COVID is a term used to describe the persistence or the development of new symptoms that can occur after an acute infection. Little is known about the association between arterial stiffness and Long COVID. An observational, cross‐sectional study in which arterial stiffness was measured with pulse wave velocity (PWV) was carried out in 74 participants between 19 and 40 years old (53 with Long COVID, 21 age and gender‐matched controls). Data was collected from participants between 1 and 9 months after acute COVID‐19 infection using the Complior analyze unit protocol. The Long COVID group had higher carotid‐radial‐PWV (crPWV) than controls (10 m/s interquartile range [IQR] 8.5–11.2 m/s) versus 8.8 m/s (IQR 7.7–9.2 m/s) as was their carotid‐radial‐arterial stiffness index (crASI) (2.26 cm/ms (IQR 1.9–2.56 cm/ms) vs. 2.01 cm/ms (IQR 1.82–2.27 cm/ms); *p* < 0.05) in both. They also had more type‐A waveforms, indicating increased arterial stiffening. Peripheral arterial stiffness was higher in adults with Long COVID than in controls who were never infected with SARS‐CoV‐2 as noted by the elevated levels of crPWV and crASI among adults with Long COVID.

## Graphical abstract

Pulse wave velocity (PWV) (carotid‐radial (cr) and carotid‐femoral (cf)) with their Arterial Stiffness indexes (ASI) were obtained using the Complior Analyze Unit protocol. The LLong COVID participants had higher crPWV and crASI compared to the control group who had never tested positive for COVID‐19. Persons with Long COVID also showed augmented waveforms, typical of stiffened arteries. crPWV, carotid–radial pulse wave velocity; crASI, carotid–radial arterial stiffness index; cfPWV, carotid‐femoral pulse wave velocity; cfASI, carotid‐femoral arterial stiffness index. Created with BioRender.com.

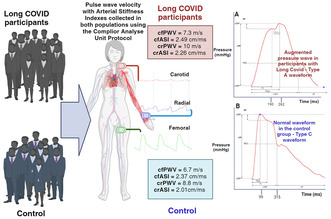

## 1 INTRODUCTION

Long COVID is defined as the persistent state of symptoms that occur in individuals and lasts for at least 4–12 weeks after an acute infection with severe acute respiratory syndrome coronavirus‐2 (SARS‐CoV‐2) (Health NIf, and Excellence C, 2021; World Health Organisation, 2024). It is characterized by various symptoms such as fatigue, headaches, hair loss, dyspnea, joint and muscle pain, and initiation of hypertension (Delalic et al., 2022; Kamal et al., 2021). Though the underlying pathophysiology is unknown, people with heart disease, diabetes, obesity, cancer, and kidney disease have a higher risk of developing Long COVID (Ayoubkhani et al., 2021; Giannis et al., 2021), endothelial dysfunction is one of the most cited contributing factors to the disease mechanism. Further, the dysfunction of the endothelium is associated with risk factors such as persistent inflammation, increased oxidative stress, hypercoagulation, and organ damage (Colling & Kanthi, 2020; Froldi & Dorigo, 2020; Libby & Luscher, 2020), which are factors that are prevalent in Long COVID.

Arterial stiffness is a phenomenon highly linked to mechanisms that result from aging, and injury to the vasculature and more directly the endothelial cells. The endothelium regulates blood flow and blood pressure by producing vasoactive substances such as nitric oxide, prostacyclin, endothelin‐1, and platelet‐activating factor (De Caterina & Libby, 2007; Green, 2020). When the endothelium is damaged, the bioavailability of nitric oxide is reduced, which increases the risk of hypertension, thrombosis, and cardiovascular disease (Stanhewicz & Kenney, 2017; Torres‐Narvaez et al., 2019; Tousoulis et al., 2012). Specifically, nitric oxide prevents the virus from entering cells by blocking the ACE‐2 receptor, which the virus uses to infect cells in the lungs and blood vessels (Hamming et al., 2004), further enhancing the spread of the virus and its effects. The virus also causes large amounts of inducible nitric oxide synthase to be produced in SARS‐CoV‐2 infection patients (Gelzo et al., 2022) leading to overproduction of nitric oxide which adds to the process of damaging the endothelial cells. It binds with superoxides which are a product of oxidative stress triggered by hyperinflammation that activates NADPH oxidase triggered by COVID‐19 (De Caterina & Libby, 2007).

The non‐enzymatic reaction of superoxides and nitric oxide forms peroxynitrite (Forcados et al., 2021) and this reaction is non‐enzymatic (Aktan, 2004; Huie & Padmaja, 1993). Peroxynitrite has a targeted effect on endothelial cells, as it partially penetrates the cell membranes via anion channels (Denicola et al., 1998; Szabo et al., 2007) and specifically influences the fragmentation of cell DNA, which leads to endothelial cell dysfunction (Denicola et al., 1998). It also induces apoptosis and poly (ADP‐ribose) polymerase (PARP)‐dependent cell death (Dickhout et al., 2005), leading to a reduction in vascular compliance (Mihm et al., 2000). Additionally, Long COVID can exacerbate prolonged inflammation (Maltezou et al., 2021) which may lead to a loss of arterial flexibility, further damaging the inner layer of the arteries (Hoffmann et al., 2020; Laurent et al., 2006). This reduction in compliance is known as arterial stiffness and seems to worsen after SARS‐CoV‐2 infection, even if the initial symptoms are mild (Szeghy et al., 2022) causing increased cerebrovascular disease and general fragility of blood vessels (Bianchi et al., 2021; Mao et al., 2020).

Worldwide, persistent symptoms of Long COVID include cough, fatigue, and shortness of breath (Carfi et al., 2020; Goertz et al., 2020; Sperling et al., 2022; Tenforde et al., 2020). While studies in China report persistent abnormalities in lung function and reduced gas exchange as one of the most common persistent symptoms (Blanco et al., 2021; Mo et al., 2020; Zhao et al., 2020), sub‐Saharan Africa reports lingering chest pain and cough independent of age, gender, or hypertension before SARS‐CoV‐2 infection (Osikomaiya et al., 2021). In addition to these observations, countries such as Zambia have cited that the presence of co‐morbidities prior to acute infection increases the risk of developing Long COVID (Zulu et al., 2022). Currently, Long COVID is now over 41% prevalent in Africa (Nyasulu et al., 2023; Osikomaiya et al., 2021; Zulu et al., 2022) with ~17% of persons with acute SARS‐CoV‐2 infection reporting symptoms approximately 2 months post‐acute SARS‐CoV‐2 infection in 2022 (Zulu et al., 2022). This number has increased to 28% by 2023 (Malambo et al., 2024). With such a rate of increase in the number of persons with Long COVID and the probable role of arterial stiffness, it is imperative to understand the current state of the events. We hypothesized that arterial stiffness would be significantly higher in adults with Long COVID than in healthy adults who have never tested positive for COVID‐19.

## 2 METHODS

### 2.1 Study population

This observational, cross‐sectional study, was conducted in Lusaka at the Lusaka Apex Medical University in Lusaka, Zambia, between the years 2023 and 2024. We enrolled adults from the ages of 18–40 years, male and female. Participants were carefully selected from a homogeneous socio‐economic and geopolitical background to maintain consistency across the study. Standardized questionnaires were randomly distributed to collect data on post‐SARS‐CoV‐2 infection manifestations. The questionnaire was divided into the collection of demographic data, SARS‐CoV‐2 infection data, other comorbidities, and data on post‐SARS‐CoV‐2 infection acute recovery manifestations. The study was approved by The Lusaka Apex Medical University Board of Research Ethics Committee (LAMUBREC–FWA00000338; IRB00001131 of IORG0000774). Regulatory approval was also obtained from the National Health Research Authority. All subjects signed consent forms before data collection.

The inclusion criteria for the test group included people who self‐reported to have tested positive for COVID‐19, with either polymerase chain reaction or antigen tests, at least 4 months before enrolment in the study and no earlier than May 2020. Acute infection ranged from mild to severe, characterized by fever, malaise, cough, upper respiratory symptoms, and/or less common features of SARS‐CoV‐2 infection in the absence of dyspnea (shortness of breath). Participants were only included if their recall period was long enough to potentially have a symptom for at least 4 weeks. Participants with diagnoses made before May 2020, with hypertension, diabetes mellitus, or respiratory diseases such as bronchitis or asthma, pregnant women, smokers, and people with self‐reported diagnosed infections were excluded.

Participants who reported suffering from post‐COVID symptoms for less than 1 month were also excluded. Only participants who had not previously tested positive for COVID‐19 were eligible for the control group. Exclusion criteria included hypertensives, diabetics, people with diagnosed respiratory diseases such as bronchitis or asthma, pregnant women, smokers, people with self‐reported diagnosed infections, and people who suspected that they had contracted COVID‐19 but had not been tested. The controls were selected in a 1:3 ratio of controls to positives.

We defined Long COVID as symptoms lasting for a month or longer, in line with the Center for Disease Control and Prevention (CDC) definition of the time frame Long COVID (Centers for Disease Control and Prevention, 2024).

### 2.2 Data collection protocol

Data for arterial stiffness was collected in a controlled room temperature environment to maximize participant comfort. Participants were re‐familiarized with the protocol on arrival before signing a printed consent form. This was the second screening stage and participants only consented if they met the inclusion criteria. Information on risk factors for non‐communicable diseases and general socio‐demographic data was collected using the World Health Organization (WHO) STEPS survey tool. Height and weight were measured using a Micro T3 PW‐BMI digital scale. Blood pressure was measured using a HEM‐757 blood pressure monitor (Omron, Kyoto, Japan) in sitting and supine positions.

### 2.3 Arterial stiffness measurement

To assess arterial stiffness as a marker of vascular dysfunction, we used both carotid‐radial and carotid‐femoral pulse wave velocities (crPWV and cfPWV) and their respective arterial stiffness indexes (crASI and cfASI) to capture both peripheral and central arterial stiffness, respectively. crPWV and cfPWV were measured using the Complior analyze unit (V1.9 Beta version 2013; ALAM‐Medical, Saint‐Quentin‐Fallavier, France) as described in our previous studies (Chikopela et al., 2021; Kaluba et al., 2021).

Standing height (without shoes) was measured using a Micro T3 PW‐BMI digital medical scale and entered manually into the Complior analyze unit (V1.9 Beta version 2013; ALAM‐Medical, Saint‐Quentin‐Fallavier, France). For this purpose, the height (in centimeters) was divided by the time between the peaks of the transit time of the pulse waveform (in milliseconds) to obtain the ASI in cm/ms.

### 2.4 Statistical analysis

The sample size was based on a study by Oikonomou et al. (2023) who measured cfPWV in 30 participants with 34 age‐matched controls without Long COVID. With a standard deviation of 2.31. Since the actual difference in cfPWV means between those who had Long COVID and did not have Long COVID was 2.1 m/s, only 43 subjects who had Long COVID and 15 control subjects were required to reject the null hypothesis that the population means of the subjects had Long COVID and the control groups are equal, with a power of 0.85. The Type I error probability associated with this test of the null hypothesis was 0.05.

Demographic and clinical variables for Long COVID versus the control group were reported in numbers and percentages for categorical variables and means (with standard deviations (SD)) or medians (with interquartile ranges (IQR)) were reported for continuous variables. Differences between cases and controls were tested with a Fisher's Exact test for categorical variables (proportions), a Student's *t*‐test for normally distributed continuous variables (means), or an independent nonparametric Mann–Whitney *U*‐test for continuous variables that may not be normally distributed. The normality of the outcome data was assessed using the Shapiro–Wilk test. PWV was also compared across the severity of acute‐COVID groups (mild, moderate, and severe) using Analysis of Variance (ANOVA). The ANOVA was followed by the Bonferroni post‐hoc test where appropriate to assess pairwise comparison using STATA software and presented as mean ± SD. In all cases, *p* < 0.05 was deemed statistically significant.

Waveform analysis involved estimating the mean waveform indexes by dividing the augmentation indexes (Aix) by the pulse pressure. This provided numbers that could be used to determine the type of waveform (A—ratio equal to or greater than 0.12, B—ratio between 0 and 0.12, and C—ratio less than 0). Comparisons were made between groups to determine which waveform was predominant and Fisher's Exact Test was used to determine significant differences at a *p*‐value of 0.05. A within‐group analysis was also performed to determine Aix differences in cfPWV waveforms and crPWV waveforms in the two populations. Seventy‐four representative waveforms were analyzed, 53 from the Long COVID group and 21 from the control group. A two‐sided contingency table analysis was performed to determine whether the type of waveform was associated with the two groups of interest with a two‐sample proportion test calculator used to compare column proportions.

From the total number of 74 participants, we fit a multiple linear regression to control for a maximum of five predictor variables (74/15) (Harrell, 2015). The independent variable Long COVID status was adjusted for age, sex, and weight, and the time the participant last tested positive for SARS‐CoV‐2 was used to predict the dependent variables (cfPWV or crPWV). The pooled data (both Long COVID and control groups) were used to adjust for the said independent variables. The covariates selected a priori based on the published risk factors for arterial stiffness were age, sex, and obesity (Ye et al., 2016). The models were constructed stepwise, adjusting for variables to note which model had the best associative relationship with the outcome variable. The models with the highest *R* ^2^ (the proportion of the variance for a dependent variable that is explained by an independent variable). The *β* coefficients were used to indicate the degree of change in the outcome in response to a unit change of the inference variable.

All measurements were physically transferred to data collection sheets, signed by the investigators, and then moved to the data analysis package STATA version 15 (Stata Corp, University Station, Texas, United States of America) and GraphPad Prism version 10.0.0 for Windows, GraphPad Software, Boston, Massachusetts USA, www.graphpad.com.

## 3 RESULTS

### 3.1 Demographic and clinical characteristics

We recruited 74 participants, 53 (27 female; 26 male) with long COVID and 21 (11 female; 10 male) controls, aged between 19 and 40 years. The participants with Long COVID had a duration of 1–9 months of persistent symptoms. Participant demographics and baseline values were comparable to those of participants without Long COVID, except for weight. The participants with Long COVID weighed significantly more than the control group, as highlighted in Table 1. We used medians for age, weight, central SBP and central pulse pressure, crPWV, crASI, cfPWV, and cfPWV as the data were skewed, and we used mean values for BMI, weight, central DBP, and MAP as the data were evenly distributed.

**TABLE 1:** Demographic and clinical characteristics of the studied population.

Parameter	Long COVID *n* = 53	Control *n* = 21	*p*
Population size *n* (%)	53 (72%)	21 (28%)	
Sex (female)	27 (51%)	11 (52%)	0.911
Age (years)	25 [24–26]	24 [23–25]	0.147
BMI (kg/m^2^)	23.7 ± 3.91	21.6 ± 4.50	0.060
Height (cm)	169.9 ± 10.18	164.9 ± 8.79	0.050
Weight (kg)	66 [60–78]	59 [51–64]	**0.007** *
Heart rate (bpm)	69 [62–77]	69 [62–87]	0.490
cSBP (mmHg)	111 [104–124]	118 [101–131]	0.976
cDBP (mmHg)	79 ± 8.23	79 ± 7.96	0.887
cMAP (mmHg)	91.6 ± 9.10	90.7 ± 8.56	0.692
cPP (mmHg)	30 [25–47]	33 [22–49]	0.952
Former smokers *n* (%)	4 (8%)	1 (5%)	0.667
Alcohol consumption *n* (%)	37 (70%)	13 (62%)	0.512
Exercise–yes *n* (%)	43 (81%)	18 (86%)	0.747

*Note*: Values are in percentage, mean (±SD); and in case of a skewed distribution of data, medians [interquartile ranges [IQR]].

Abbreviations: BMI, body mass index; cDBP, central diastolic blood pressure; cMAP, central mean arterial pressure; cPP, central pulse pressure; cSBP, central systolic blood pressure.

* *p* < 0.05.

### 3.2 Persistent symptoms in Long COVID group

Long COVID participants reported a total of 12 persistent symptoms (Table 2), of which fatigue (*n* = 20), cough (*n* = 25), and persistent headache (*n* = 24) were the most common symptoms. Compared to males, the females reported experiencing almost twice as much fatigue, chest pain, and breathing difficulties. As shown in Table 2, females also reported to be experiencing more mild energy loss compared to males.

**TABLE 2:** Persistent COVID‐19‐related symptoms in the Long COVID group.

Symptom	*n* (%)	Males (*n*)	Females (*n*)
Fatigue	20 (38)	7	13
Cough	25 (47)	13	12
Headache	24 (45)	12	12
Breathing difficulties			
Mild	7 (13)	2	5
Moderate	7 (13)	2	5
Palpitations	12 (23)	6	6
Chest pain	14 (26)	4	10
General body aches	13 (25)	7	6
Sore throat	10 (19)	5	5
Loss of smell or taste	15 (28)	9	6
Energy loss			
Mild	9 (17)	1	8
Moderate	1 (2)	0	1
Severe	2 (4)	0	2
Mobility difficulties			
Mild	5 (9)	2	3
Moderate	2 (4)	1	1
Severe	5 (9)	2	3
Weight loss	2 (4)	1	1

*Note*: Values are *n* (%); *n*, number of participants, and % is the percentage.

### 3.3 Arterial stiffness comparison in Long COVID and control

The two cohorts had comparable medians of cfPWV and cfASI. crPWV was higher in participants with Long COVID with a value of 10 m/s (IQR 8.5–11.2 m/s) and 8.8 m/s (IQR 7.7–9.2 m/s) in participants in the control group (*p* < 0.05), as highlighted in Table 3. Additionally, crASI was higher in participants with Long COVID (2.26 cm/ms (IQR 1.9–2.56 cm/ms)) compared to (2.01 cm/ms (IQR 1.82–2.27 cm/ms)) observed in participants in the control group (*p* < 0.05). The Coefficient of Variation was 14% for cfPWV and 8% for crPWV both calculated as (SD ± Mean) × 100%.

**TABLE 3:** Arterial stiffness variables by Long COVID status.

Variables	Long COVID (*n* = 53)	Control (*n* = 21)	*p*
crPWV (m/s)	10 [8.5–11.2]	8.8 [7.7–9.2]	0.019*
crASI (cm/ms)	2.26 [1.9–2.56]	2.01 [1.82–2.27]	0.045*
cfPWV (m/s)	7.3 [6.4–9.2]	6.7 [5.6–7.8]	0.1202
cfASI (cm/ms)	2.49 [2.22–3.06]	2.37 [1.99–2.7]	0.207

*Note*: Values are displayed as medians [interquartile ranges [IQR]].

Abbreviations: cfASI, carotid‐femoral arterial stiffness index; cfPWV, carotid‐femoral pulse wave velocity; crASI, carotid–radial arterial stiffness index; crPWV, carotid–radial pulse wave velocity.

* *p* < 0.05.

**FIGURE 1:**
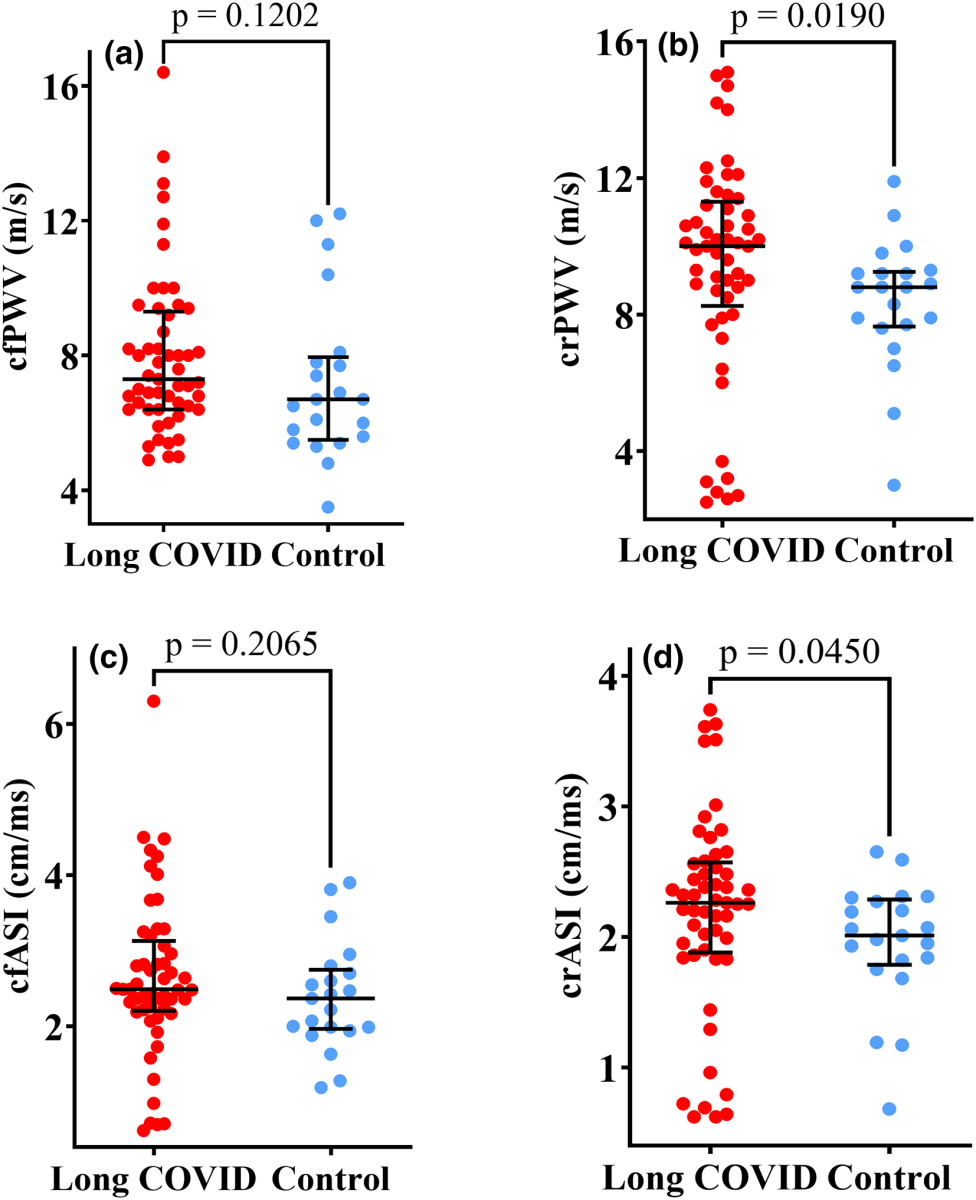
The top panel shows pulse wave velocity (cfPWVand crPWV) and the bottom panel is a comparison of Arterial Stiffness Indexes (cfASI and crASI) between 53 participants with Long COVID and 21 controls. There was no significant difference in cfPWV (A) between participants with Long COVID and the Control group. crPWV (B) was significantly higher in the participants with Long COVID (10 m/s (IQR 8.5–11.2 m/s)) compared to the control (8.8 m/s (IQR 7.7–9.2 m/s); *p* = 0.0190). There was no significant difference in cfASI (C) between participants with long COVID and the Control group while crASI (D) was significantly higher in the participants with Long COVID (crASI of 2.26 cm/ms (IQR 1.9–2.56 cm/ms)) compared to controls, whose median was 2.01 cm/ms (IQR 1.82–2.27 cm/ms); *p* = 0.0450. We used the Mann–Whitney *U*‐test for skewed data to note the statistical differences (*p* = 0.05). The scatter dot plots show the median (middle lines) with interquartile ranges (minimum to maximum values). crPWV, carotid–radial pulse wave velocity; crASI, carotid–radial arterial stiffness index; cfPWV, carotid‐femoral pulse wave velocity; cfASI, carotid‐femoral arterial stiffness index.

Both crPWV and crASI had lower medians in the control group than in the participants with long COVID. Participants with Long COVID had a numerically higher median value of cfASI of 2.49 cm/ms (IQR 2.22–3.06 cm/ms) compared to controls, whose median was 2.37 cm/ms (IQR 1.99–2.7 cm/ms), though not statistically significant. As shown in Figure 1, this was also noted in cfPWV which was numerically higher among participants with Long COVID than the control group without statistical significance.

### 3.4 Symptom severity and outcome

There was a significant association between the severity of acute COVID‐19 and arterial stiffness as measured by crPWV and crASI as shown in Table 4. Mild symptoms during acute COVID‐19 infection were significantly associated with increased crPWV and crASI in comparison to moderate symptoms with *p*‐values of 0.026 and 0.041, respectively.

**TABLE 4:** Arterial stiffness and severity of acute COVID‐19 infection.

Acute COVID‐19 infection	cfPWV (m/s)	cfASI (cm/ms)	crPWV (m/s)	crASI (cm/ms)
Mild (Froldi & Dorigo, 2020)	9.0 ± 2.31	2.84 ± 1.125	12.1 ± 1.92^a^	2.79 ± 0.593^a^
Moderate (Libby & Luscher, 2020)	8.2 ± 2.86	2.78 ± 1.328	7.8 ± 3.77^b^	1.82 ± 0.888^b^
Severe (Zulu et al., 2022)	7.7 ± 2.31	2.57 ± 1.017	9.2 ± 3.06^a,b^	2.14 ± 0.730^a,b^
*p*‐value	0.372	0.761	0.021*	0.033*

*Note*: Post ANOVA any variables with significant *p*‐values had a, b, and c used after the Bonferroni test for pairwise comparisons to note differences among means (± SD). * *p* < 0.05.

### 3.5 Effect of sex on arterial stiffness

#### 3.5.1 Arterial stiffness comparison in the study population by sex

**FIGURE 2:**
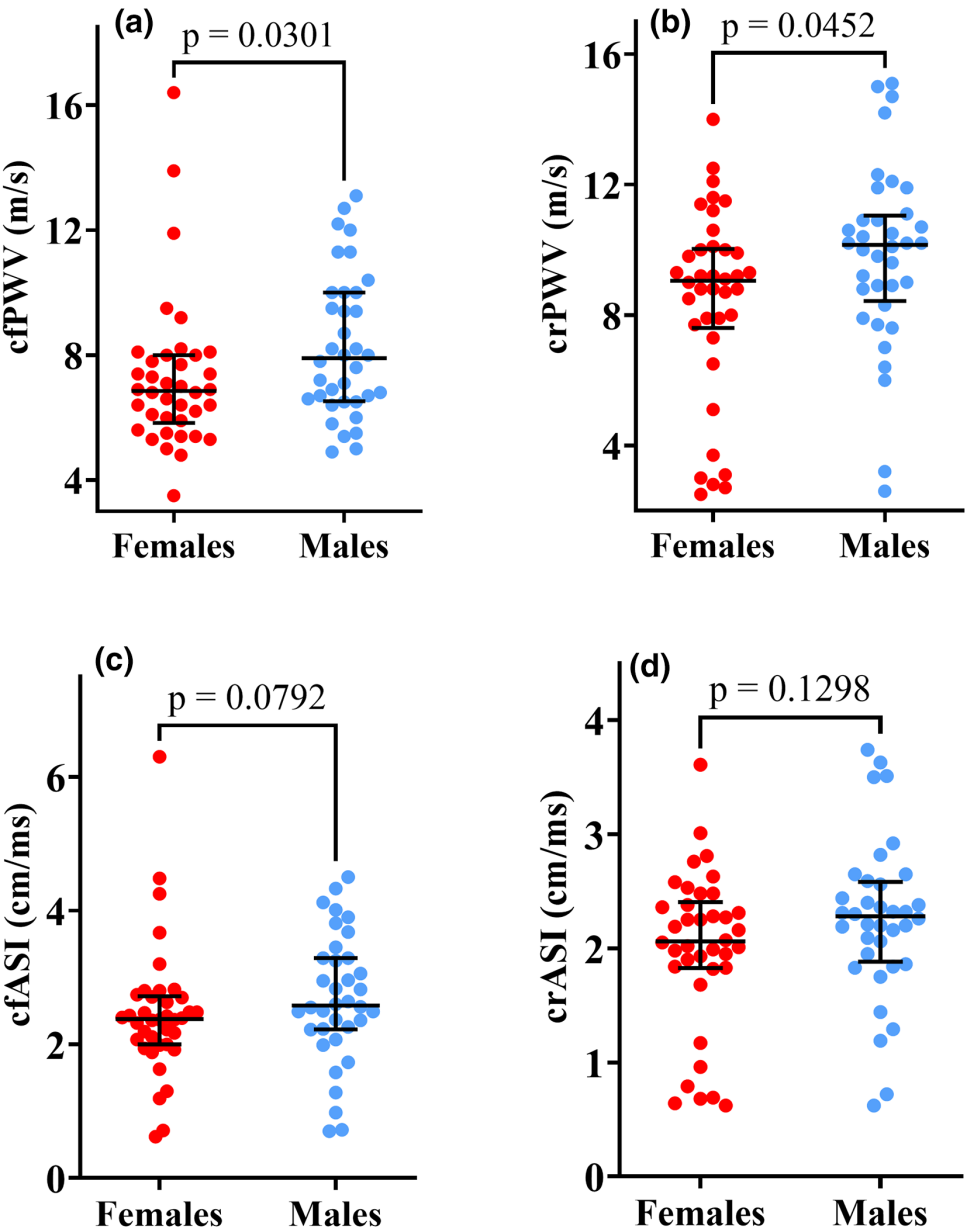
Arterial stiffness in the general population stratified by gender with 38 females and 36 males. Top panel A shows that the males had a significantly higher cfPWV of 7.9 m/s [IQR 6.5–10.0 m/s] compared to the females (6.9 m/s [IQR 5.8–8.0 m/s]); *p* = 0.030. The crPWV was also higher in the male participants compared to the females (10.2 m/s [8.4–1.1 m/s] vs. 9.1 m/s [IQR 7.6–10.0 m/s]); *p* = 0.045 (Top panel B). There were no significant differences in arterial stiffness indexes between males and females (Bottom panel). We used the Mann–Whitney *U*‐test for skewed data to note the statistical differences (*p* = 0.05). The scatter dot plots show the median (middle lines) with interquartile ranges (minimum to maximum values). crPWV, carotid–radial pulse wave velocity; crASI, carotid–radial arterial stiffness index; cfPWV, carotid‐femoral pulse wave velocity; cfASI, carotid‐femoral arterial stiffness index.

In the study population, females showed a significantly lower cfPWV 6.9 m/s (IQR 5.8–8.0 m/s) compared to males 7.9 m/s (6.5–10.0 m/s); *p* = 0.030 as well as a lower crPWV 9.1 m/s (IQR 7.6–10.0 m/s) versus 10.2 m/s (8.4–11.1 m/s); *p* = 0.045 as depicted in Figure 2.

#### 3.5.2 Within groups by sex arterial stiffness comparison

**FIGURE 3:**
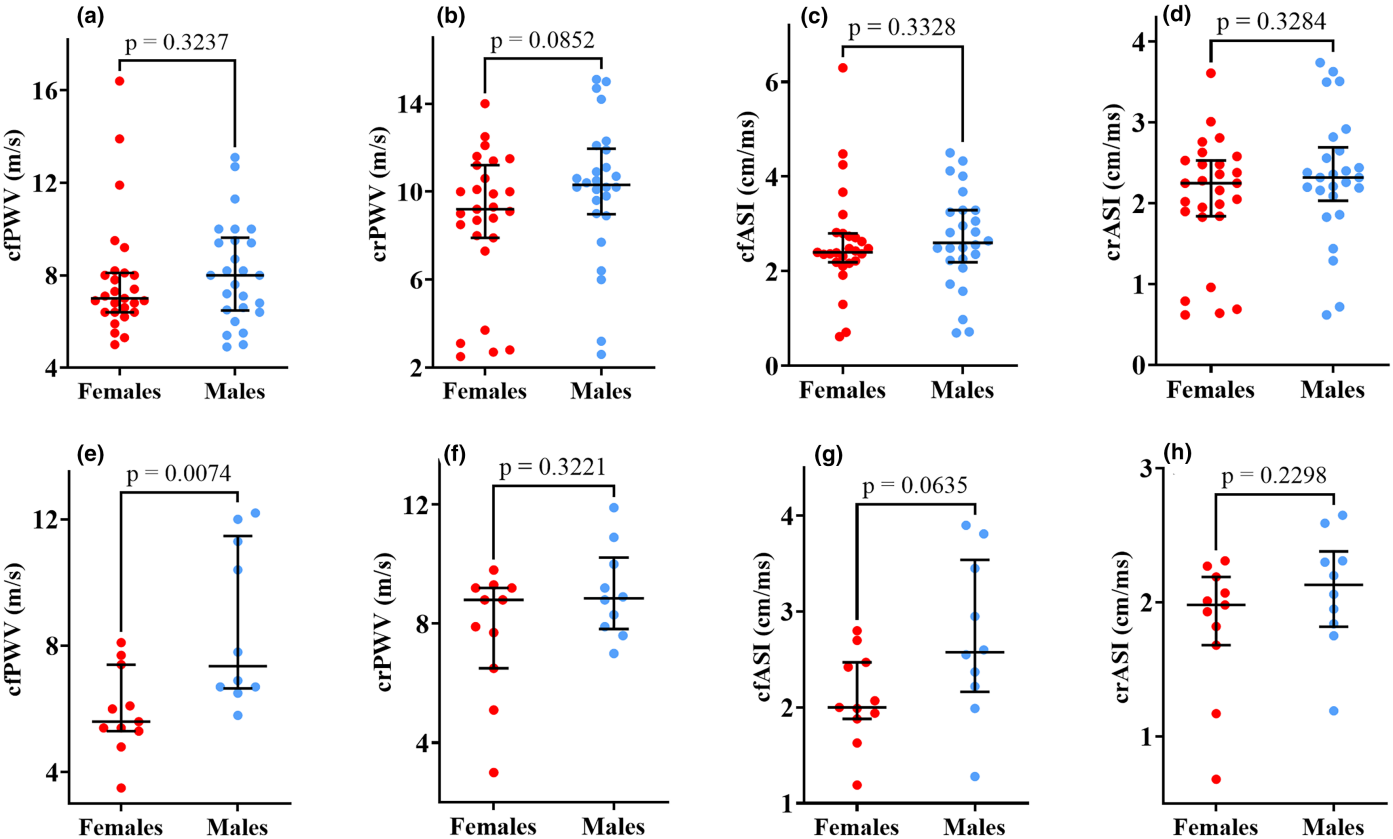
Arterial stiffness stratified by gender: The top panel is Long COVID (A–D) with 27 females and 26 males, and the bottom panel is for the Controls (E–H) with 11 females and 10 males. There was a significant difference in cfPWV between females and males in the control group. We used the Mann–Whitney *U*‐test for skewed data to note the statistical differences (*p* = 0.05). The scatter dot plots show the median (middle lines) with interquartile ranges (minimum to maximum values). crPWV, carotid–radial pulse wave velocity; crASI, carotid–radial arterial stiffness index; cfPWV, carotid‐femoral pulse wave velocity; cfASI, carotid‐femoral arterial stiffness index.

Within the control group, the males had higher cfPWV compared to the females 7.4 m/s (IQR 6.7–11.5 m/s) versus 5.6 m/s (IQR 3.5–7.4 m/s); *p* = 0.007 (Figure 3).

#### 3.5.3 Within sex by group arterial stiffness comparisons

**FIGURE 4:**
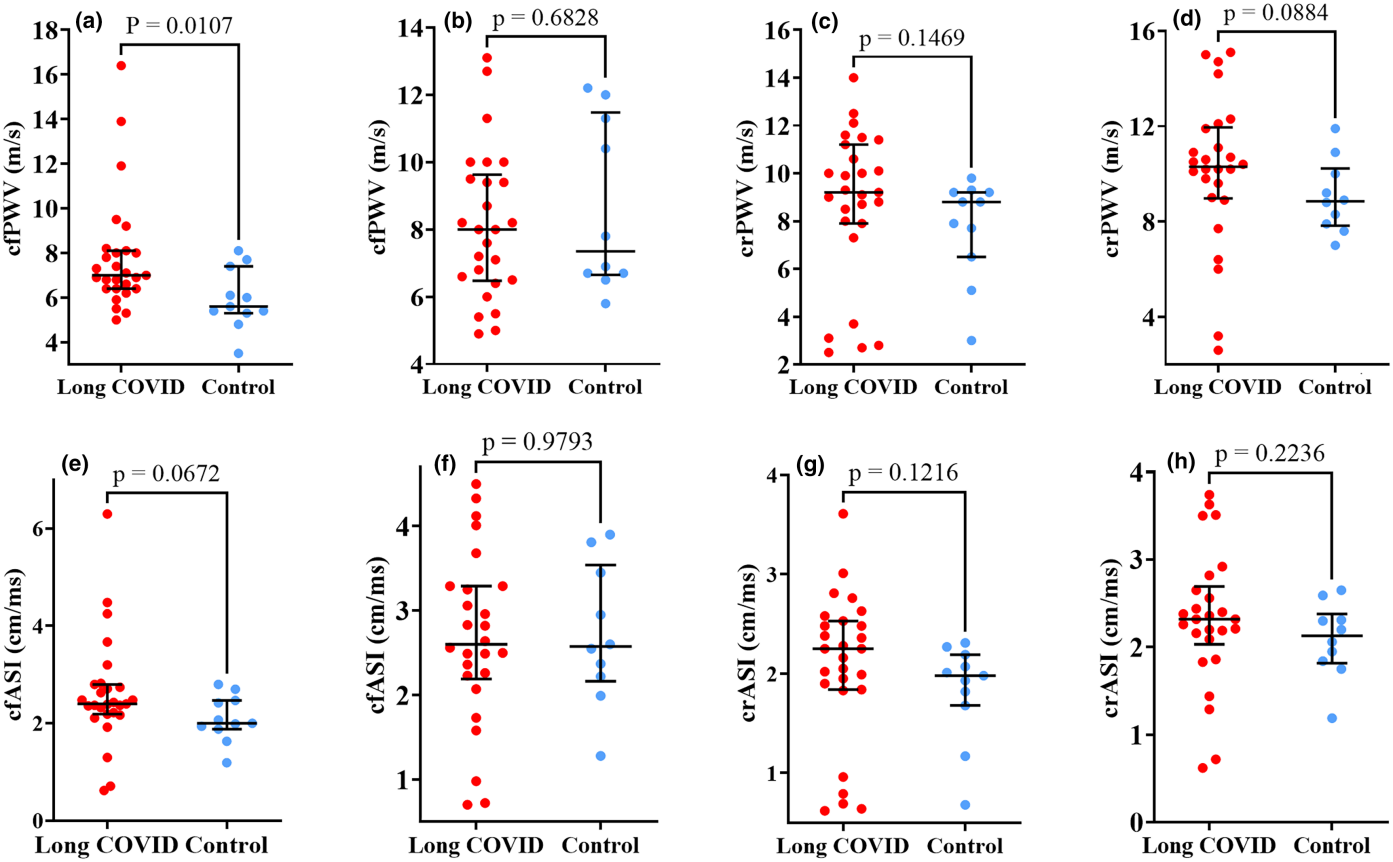
Arterial stiffness stratified by Long COVID status: (A, C, E, G) are comparisons within females (27 with Long COVID and 11 controls) while (B, D, F, H) are comparisons within males (26 with Long COVID and 10 controls). There was a significant difference in cfPWV between Long COVID and control groups among females (a). We used the Mann–Whitney *U* test for skewed data to note the statistical differences (*p* = 0.05). The scatter dot plots show the median (middle lines) with interquartile ranges (minimum to maximum values). crPWV, carotid–radial pulse wave velocity; crASI, carotid–radial arterial stiffness index; cfPWV, carotid‐femoral pulse wave velocity; cfASI, carotid‐femoral arterial stiffness index.

A sub‐analysis within females showed that cfPWV was higher among females with Long COVID at 7.0 m/s (IQR 5.0–8.1 m/s) compared to the control who had a median cfPWV of 5.6 m/s (IQR 3.5–7.4 m/s) with a *p*‐value of 0.011 (Figure 4).

Compared to males and while holding Long COVID status constant, females were associated with a lower cfPWV and crPWV of approximately 1 m/s (CI of −2.08 to 0.09); *p* = 0.07 and 1.38 m/s (CI of −2.69 to −0.06); *p* = 0.04, respectively.

### 3.6 Arterial stiffness by waveform analysis

**FIGURE 5:**
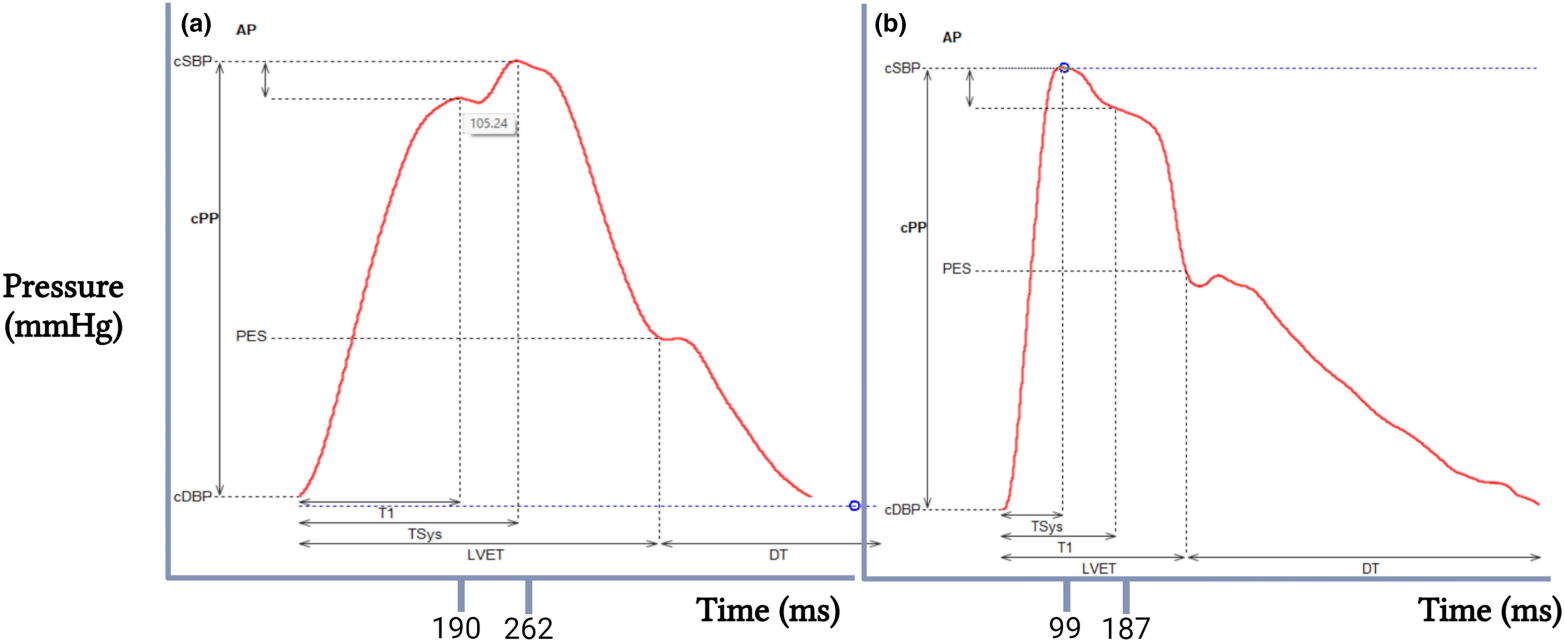
Extract of an example of type A waveform (a) from a Long COVID participant and type C waveform (b) from the control group. Type A waveform has a rapid rise to peak pressure (P1–105 mmHg) due to left ventricular ejection, followed by a higher systolic pressure peak (105 mmHg) due to an early systolic reflected wave that increases systolic pressure and augmentation pressure (3 mmHg). The augmentation index (Aix), calculated as 100 × (3 mmHg/32 mmHg), is 8.52%, indicating an early reflection before completion of the systolic wave and a secondary late systolic peak in early diastole (108 mmHg). The Type C waveform shows a steep, uninterrupted rise in forward wave pressure to its first systolic peak (114 mmHg) from the base of the wave caused by left ventricular ejection. The reflective wave adds to the forward wave in late systole or early diastole. The Aix was characterized as less than 0% (i.e., 100 × (−3 mmHg/32 mmHg) = −9%). The timing of the reflected wave was completed before the timing of the systolic wave had elapsed. It also shows a second late systolic shoulder between the first peak incisura and a second pressure rise in early diastole. cSBP, central systolic blood pressure; cPP, central pulse pressure; cDBP, central diastolic blood pressure; AP, augmentation pressure; PES, end‐systolic blood pressure; Tsys, the timing of the systolic wave; T1, timing of the reflected wave; LVET, left ventricular ejection time; DT, diastolic time; P1, first systolic peak; P2, second systolic peak.

The Long COVID participants had significantly more type A waveforms, while the control group had more type C waveforms (see Table 5 and a representative waveform in Figure 5). A Fisher's exact test showed statistically significant differences in the association between some waveform types and Long COVID status, with a *p*‐value of 0.002. Pairwise comparisons showed a significant difference in the number of associations between waveforms A and C with Long COVID status (*p* = 0.001). There was a significantly higher association of waveform A with being in the Long COVID group compared to waveform C which was associated with being not having Long COVID.

**TABLE 5:** Waveform characteristics comparing participants with Long COVID and the control group.

Waveform types	Long COVID	Control group	*p*
Type A (%)	**31 (59%)**	**4 (19%)**	
Type B (%)	**7 (13%)**	**2 (2%)**	
Type C (%)	**15 (28%)**	**15 (71%)**	
Augmentation Index			
cfPWV–Type A	84.8 ± 27.87	83.8 ± 27.87	0.955
cfPWV–Type B	18.23 ± 13.78	14.7 ± 14.19	0.616
cfPWV–Type C	−30.0 ± 29.20	−18.20 ± 16.93	0.243
crPWV–Type A	78.1 ± 30.25	73.3 ± 41.51	0.847
crPWV–Type B	15.2 ± 13.14	15.5 ± 11.67	0.969
crPWV–Type C	−19.7 ± 15.16	−24.2 ± 18.78	0.456

Abbreviations: cfASI, carotid‐femoral arterial stiffness index; cfPWV, carotid‐femoral pulse wave velocity; crASI, carotid–radial arterial stiffness index; crPWV, carotid–radial pulse wave velocity. Bold indicates significance level at *p* < 0.05.

Analysis of the waveforms and their Aix which are specific to cfPWV and crPWV, showed no significant difference between the waveforms of cfPWV and crPWV. Type A waveforms were higher in both cfPWV and crPWV in the Long COVID population, as was type B in cfPWV, as shown in Table 5.

### 3.7 Regression analysis for arterial stiffness

A regression analysis taking into account gender, weight, and age, as well as an interaction between age and gender, there was no significant effect of Long COVID status on crPWV. The analysis explained 19% of the variance in crPWV by a combination of these factors. It showed a significant influence of crPWV; *F* (5, 68) = 3.23, *p* = 0.011. The final association model showed that having Long COVID was associated with a 1.39 m/s increase in crPWV as summarized in Table 6.

**TABLE 6:** Multiple linear regression of arterial stiffness and Long COVID.

Variable	crPWV			crASI		
	*β* coef	CI	*p*	*β* coef	CI	*p*
Long COVID (yes)	1.385	−0.962 to 2.868	0.066	0.334	−0.029 to 0.65	0.070
Weight (kg)	−0.027	−0.075 to 0.022	0.281	−0.007	−0.019 to 0.005	0.244
Sex (female)	9.186	−1.777 to 19.75	0.100	2.100	−0.514 to 4.714	0.124
Age (years)	−0.069	−0.287 to 0.150	0.533	−0.021	−0.071 to 0.331	0.438
Sex#age (female)	0.445	−0.874 to −0.016	0.042*	−0.100	−0.206 to 0.002	0.061

*Note*: Model adjusted for age, gender, weight, and an interaction term between age and gender in both models.

Abbreviations: CI, 95% confidence interval; coef, coefficient; crASI, carotid–radial arterial stiffness index; crPWV, carotid–radial pulse wave velocity; Sex#age, interaction between gender and age.

* *p* < 0.05.

A regression analysis was adjusted for gender, weight, and age and an interaction between age and gender. There was no significant effect of Long COVID status when adjusted for the other covariates. The analysis explained that 17% of the variance in the crASI was due to a combination of these factors. There was a significant effect of crASI; *F* (5, 68) = 2.78, *p* = 0.024. The final association model showed that having a Long COVID increased the degree of association with arterial stiffness, as measured by crASI, by 0.33 cm/ms. crPWV = 12.52–(0.027) weight–(0.069) age + (8.99) sex + (1.386) long COVID status–(0.445) age × sex interaction; crASI = 3.07–(0.007) weight–(0.021) age + (2.052) sex + (0.334) Long COVID status–(0.100) age × sex interaction with female and presence of COVID as references in the categorical variables.

### 3.8 Regression analysis within Long COVID group

A linear regression analysis within the Long COVID group using similar predictor variables including the time the participants last tested positive did not show any significant association with arterial stiffness variables as summarized in Table 7.

**TABLE 7:** Multiple linear regression of arterial stiffness in Long COVID.

Variable	cfPWV		cfASI		crPWV		crASI	
	*β* coef	CI	*β* coef	CI	*β* coef	CI	*β* coef	CI
Weight (kg)	0.01	−0.037 to 0.1	0.001	−0.02 to 0.02	−0.01	−0.07 to 0.05	−0.003	−0.02 to 0.01
Age (years)	0.08	−0.20 to 0.37	0.04	−0.09 to 0.16	−0.24	−0.59 to 0.10	−0.055	−0.14 to 0.03
Last positive	−0.02	−0.09 to 0.04	−0.01	−0.04 to 0.02	−0.07	−0.14 to 0.01	−0.014	−0.03 to 0.004
Sex (female)	3.36	−9.76 to 16.5	3.99	−1.72 to 9.72	3.62	−12.21 to 19.5	0.854	−3.04 to 4.75
Sex#age (female)	−0.14	−0.66 to 0.38	−0.16	−0.39 to 0.06	−0.24	−0.86 to 0.39	−0.053	−0.21 to 0.10

*Note*: Models adjusted for age, gender, weight, last time test positive, and an interaction term between age and gender in both models. cfPWV model: *R* ^2^ = 0.03; F (5, 47) = 0.29; *p* = 0.918. cfASI model: *R* ^2^ = 0.07; F (5, 47) = 0.68; *p* = 0.645. crPWV model: *R* ^2^ = 0.22; F (5, 47) = 2.64; *p* = 0.035. crASI model: *R* ^2^ = 0.18; F (5, 47) = 2.07; *p* = 0.086.

Abbreviations: cfASI, carotid‐femoral arterial stiffness index; cfPWV, carotid‐femoral pulse wave velocity; CI, 95% confidence interval; coef, coefficient; crASI, carotid–radial arterial stiffness index; crPWV, carotid–radial pulse wave velocity; Sex#age, the interaction between gender and age.

## 4 DISCUSSION

In this study, we show that patients with Long COVID had increased peripheral arterial stiffness (crPWV and crASI) compared to healthy controls, while central arterial stiffness did not differ between groups as measured by cfPWV and cfASI. We further demonstrate that females seem to be more sensitive to variations in central arterial stiffness as females with Long COVID had significantly higher cfPWV compared to the control group. Additionally, individuals with Long COVID showed type A waveforms, which are characteristic of arterial stiffness (Hughes et al., 2013; Murgo et al., 1980). We also show that Long COVID appears to have a more collective association with arterial stiffness after adjustment for age, gender, and weight.

The observed significant difference in crPWV between our two study groups is consistent with several studies that have found increased arterial stiffness in populations of individuals with Long COVID (Ikonomidis et al., 2022; Jud et al., 2021; Lambadiari et al., 2021; Nandadeva et al., 2023). It signifies a reduction in the compliance of the vasculature affecting endothelial mechano‐transduction (Zieman et al., 2005) which may result in, among other things, an inability to respond physiologically to an increase in blood pressure. Being a determinant of cardiovascular mortality, and increased arterial stiffness, this population is at risk of increased cardiovascular events (Laurent et al., 2001). Notably, an increase in crPWV of 1 m/s is associated with a 15% increased risk of cardiovascular events, mortality, and death overall (Vlachopoulos et al., 2010). In contrast to our results, adults have been shown to have a significant decrease in crPWV, 4 months after diagnosis in a study by Szeghy et al. (1985). However, they did observe an initial steady increase in both crPWV and crASI among Long COVID participants before the steady decline. This signifies the importance of following up with the progression of recovery and its impact on the arterial response to the SARS‐CoV‐2 acute infection. Such observations could result from a change from a sedentary to a more active lifestyle with time, allowing the stiffness to decrease (Tanaka, 2015). Our participants with Long COVID were mostly active with over 80% doing a form of exercise daily. Fortifying our observations of a causal dose–response relationship between symptom severity and outcome, we observed a significant increase in arterial stiffness among participants who had severe acute COVID‐19 symptoms. While other studies have reported long‐lasting pathological processes in the vasculature following mild COVID‐19 disease (Podrug et al., 2023), we observed a significantly higher association between mild symptoms during acute and arterial stiffness post‐COVID compared to participants who had moderate symptoms. This could be due to community‐based treatments that were common for individuals who had mild symptoms compared to those with moderate symptoms resulting in a reduction in the quality of care. In further studies, it would be imperative to note if there is a correlation between treatment regimens and ong COVID symptoms.

Though both cfPWV and cfASI were not significantly different between the Long COVID group and the control, the two parameters were numerically higher in participants with Long COVID. This suggests that cfPWV is likely to be elevated following infection and may remain elevated beyond 9 months post‐infection. These findings results were similar to a previous study by Nandadeva, Skow, Stephens, Grotle, Georgoudiou, Barshikar, Seo, and Fadel (Nandadeva et al., 2023) in women whose recovery time after SARS‐CoV‐2 infection was between 1 and 5 months and who showed no cross‐sectional difference in cfPWV compared to healthy controls (Nandadeva et al., 2023). This lack of difference could be attributed to the shortened observation period of the population. Other studies, in middle‐aged adults, have reported a significant change in central arterial stiffness 4 months after acute infection compared to controls (Lambadiari et al., 2021). This pattern of observations was noted in the females with Long COVID within our population who showed a significantly higher central arterial stiffness compared to the control group. This signifies that in this population, the cfPWV and cfASI were a more sensitive marker to changes in arterial compliance among females.

Further consolidating our findings, participants with Long COVID showed a significantly higher number of type A waveforms, which are characteristic of arterial stiffening with peak systolic pressure in early systole after a sharp kink point (Hughes et al., 2013; Murgo et al., 1980). This subsequently increases the pressure against which the left ventricle operates (Pagoulatou et al., 2021), elevating the backward pressure on the left ventricle during systole. Consequently increasing pressure in the left ventricle and remodeling of the left ventricle (Van Popele, 2006). Conversely, the Type C waveform describes the healthier waveform that propagates slowly and is reflected in late systole or diastole and was more frequent in the control group. Ultimately, the stiffening of the aorta leads to early end‐systolic reflexes instead of the usual diastolic reflexes.

Although not statistically significant, the Aix value was higher in Long COVID participants, which is also a good predictor of changes in the vasculature and thus a good predictor of CVDs (Kaluba et al., 2015). Similarly, our findings echoed those of Nandadeva, Skow, Stephens, Grotle, Georgoudiou, Barshikar, Seo, and Fadel (Nandadeva et al., 2023) who found no significant difference in Aix75 between the COVID and control groups. Conversely, other studies have reported a significantly higher Aix was found in people after COVID‐19 (Jud et al., 2021) and in people acutely infected with SARS‐CoV‐2 (Szeghy et al., 2022). Typically, a pulse waveform has an augmentation pressure (AP), which is the pressure between the pressure introduced into the arterial tree by the ejection of the left ventricle (systolic shoulder or P1) and the peak (the highest pressure through systole). In our population, we see this characteristic more prominent in the control than in persons with Long COVID.

Though the study was not powered to analyze for significant differences by gender, it was intriguing to note patterns of lower levels of central and peripheral arterial stiffness being exhibited in females compared to male participants in the study population. Interestingly, this disparity could be attributed to the influence of estrogen which is higher in females and associated with an increased level of nitric oxide bioavailability in the vasculature (Chambliss & Shaul, 2002). Moreover, alterations in the inflammatory profile of the vasculature may promote arterial stiffening and estrogen exhibits protective anti‐inflammatory properties, such as its ability to reduce the production of proinflammatory cytokines, including IL‐1β, IL‐6, and TNF‐α (Pernis, 2007; Youssef & Stashenko, 2017). Beyond the effects of estrogen, there is evidence for a suppressive effect of androgens on male macrophage activity that may contribute to gender differences in the inflammatory response (Giron‐Gonzalez et al., 2000; Pergola et al., 2008). In support of this notion, our results show that being female is protective against arterial stiffness when adjusted for Long COVID status.

Finally, we showed that after adjusting for gender, weight, and age, Long COVID was not statistically associated with arterial stiffness as measured by crPWV or crASI. However, it was observed that in our study, male gender and having Long COVID were more associated with a higher crPWV and crASI. Further research would allow us to find out how these relationships are represented longitudinally.

The limitations of our study include a limited number of participants. As a result, it was unlikely that we would prove significant differences in our sub‐analyses including those done by sex within the Long COVID group or control groups. As no antigen tests were done in either group, the results that are based on the persistence of symptoms can only be used to hypothesize that the arterial stiffness is associated with Long COVID. However, the persistent symptoms after SARS‐CoV‐2 infection in this group may be because they already had some degree of stiffness before infection with SARS‐CoV‐2. Therefore, the control served as a benchmark for health status, and the influence of the virus. Specifically, to isolate the influence of Long COVID, an ideal benchmark would have been persons who tested positive for SARS‐CoV‐2 but experienced no lingering symptoms. Although the protocol ensured that participants with pre‐existing conditions were excluded, current cardiovascular risks could not be identified and excluded at the time of data collection, resulting in some bias. We are aware of the limitations inherent in cross‐sectional comparisons and therefore recognize that differences between subjects may vary.

### 4.1 Strengths of the study

The two groups matched on almost all baseline variables, reducing bias, and the control group consisted of healthy adults of the same age and gender who had not tested positive for SARS‐CoV‐2. Significantly, this is the first study to report findings of arterial stiffness in Long COVID in sub‐Saharan Africa and these initial results require attention for clinical relevance.

In conclusion, arterial stiffness is a promising marker for detecting arterial stiffness in individuals with persistent SARS‐CoV‐2 infection effects. Notably, we found that there is an increase in crPWV and crASI in individuals with Long COVID 1–9 months after infection. Overall, the results of this study emphasize the likely link between Long COVID and arterial stiffness in healthy adults.

## 5 CLINICAL PERSPECTIVES

The persistent symptoms of SARS‐CoV‐2 infection can lead to endothelial damage resulting from inflammation, oxidative stress, and activation of the endothelium. With the overwhelming evidence implicating arterial stiffness in the pathogenesis of cardiovascular diseases, individuals with Long COVID are susceptible to increased cardiovascular events.

In this study, we found that there is an increased arterial stiffness in individuals with Long COVID that can be detected as early as 1–9 months after acute infection. We provide a possible link between Long COVID and arterial stiffness in healthy adults.

Monitoring for incidents of cardiovascular disease may therefore be needed beyond the acute phase of SARS‐CoV‐2 infection even in individuals not predisposed to cardiovascular risks.

## AUTHOR CONTRIBUTIONS

C.T. came up with the concept of the work, participated in data acquisition, analyzed the data, interpreted, and drafted the work. B.K. participated in the initial concept and collection of data. A.K. and C.A.S. critically revised the work and gave guidance on analysis and interpretation. The first draft of the manuscript was written by Theresa Chikopela and all authors commented on previous versions of the manuscript. All authors read and approved the final manuscript.

## FUNDING INFORMATION

This study was supported by the National Institutes of Health grants R01HL147818 and R01HL144941 (A. K.).

## CONFLICT OF INTEREST STATEMENT

No competing interests.

## ETHICS STATEMENT

The study was approved by The Lusaka Apex Medical University Board of Research Ethics Committee (LAMUBREC–FWA00000338; IRB00001131 of IORG0000774). Regulatory approval was also obtained from the National Health Research Authority (NHRA).

## DISCLAIMER

Contents are solely the responsibility of the authors and do not necessarily represent the official view of the National Institutes of Health. The funders had no role in the study design, data collection and analysis, decision to publish, or preparation of the manuscript.

## Data Availability

The authors confirm that the data supporting the findings of this study are available from the corresponding author upon reasonable request.
